# Enhancing Resilience in Canadian Military Families and Communities: A Qualitative Analysis of the Reaching In… Reaching Out and Bounce Back and Thrive! Resiliency Skills Training Programs

**DOI:** 10.3389/fpubh.2021.662313

**Published:** 2021-05-20

**Authors:** Cynthia Mikolas, Hope Winfield, Lorraine Smith-MacDonald, Ashley Pike, Chelsea Jones, Melina Lee, Jennifer Griffiths, Ryan Perry, David M. Olson, Alexandra Heber, Joanne Olson, Phillip R. Sevigny, Suzette Brémault-Phillips

**Affiliations:** ^1^Military Family Resource Centre, Canadian Forces Base Edmonton, Edmonton, AB, Canada; ^2^Heroes in Mind, Advocacy and Research Consortium (HiMARC), Faculty of Rehabilitation Medicine, University of Alberta, Edmonton, AB, Canada; ^3^Royal Canadian Chaplain Service, Canadian Armed Forces, Canada and Faculty of Rehabilitation Medicine, University of Alberta, Edmonton, AB, Canada; ^4^Faculty of Rehabilitation Medicine, University of Alberta, Edmonton, AB, Canada; ^5^Department of Occupational Therapy, Faculty of Rehabilitation Medicine, University of Alberta, Edmonton, AB, Canada; ^6^1 Field Ambulance, Canadian Forces Health Services, Canadian Forces Base Edmonton, Edmonton, AB, Canada; ^7^Faculty of Nursing, University of Alberta, Edmonton, AB, Canada; ^8^Departments of Obstetrics & Gynecology, Faculty of Medicine and Dentistry, University of Alberta, Edmonton, AB, Canada; ^9^Veterans Affairs Canada, Ottawa, ON, Canada; ^10^Department of Psychiatry, University of Ottawa, Ottawa, ON, Canada; ^11^Department of Educational Psychology, Faculty of Education, University of Alberta, Edmonton, AB, Canada

**Keywords:** military, families, resilience, well-being, program evaluation (MeSH), community, mental health—related quality of life

## Abstract

**Introduction:** A new vision of resilience and well-being for Canadian military service members (SMs), Veterans and their families has been championed by the Canadian Armed Forces (CAF) and Veterans Affairs Canada (VAC). Operationalizing this vision, which aims to support those who serve/have served and their families as they navigate life during and post-service, requires the support of service providers (SPs). Training SPs to deliver complementary resilience-training programs Reaching In… Reaching Out (RIRO; for adults working with parents of young children) and Bounce Back and Thrive! (BBT; for parents of children aged 0–8 years of age) may support this vision.

**Objective:** To assess the appropriateness of RIRO/BBT trainer training for SPs, and RIRO and BBT resilience-training for military populations and families.

**Methods:** This qualitative descriptive study involved the delivery of RIRO/BBT trainer training to SPs (*n* = 20), followed by focus groups (*n* = 6) with SPs and organisational leaders (*n* = 4). Focus groups were recorded, and data were transcribed and thematically-analysed.

**Results:** Several themes emerged: (1) RIRO/BBT trainer training enabled SPs to model resilience and deliver the resilience-training programs, (2) training was appropriate and adaptable for the CAF and SMs/CMFs, and (3) training could support the development of resilient communities.

**Discussion:** RIRO/BBT trainer training and RIRO and BBT resilience-training programs use a holistic, integrated, experiential, and community approach to resilience-building and align with CAF and VAC initiatives. Once contextualised, such programs could support resilience-building in the military context.

## Introduction

A new vision of resilience for Canadian military service members (SMs), Veterans and their families has been championed by the Canadian Armed Forces (CAF) and Veterans Affairs Canada (VAC). CAF has clearly indicated that “well-supported, diverse and resilient people and families” ((1), p. 19) are its first priority. Resilience, a process through which individuals adapt to or recover from adversity (2), enables service members (SM) to be operationally-ready for domestic and international peace-keeping and combat and disaster response missions, and for Canadian military families (CMFs) to support them. Resilience across multiple domains (i.e., physical, mental, ethical/spiritual, social/cultural, familial, financial) and environments (i.e., psychosocial work, physical work, and physical home) is therefore foundational to CAF operational readiness (3) (Table 1).

**Table 1:** Total health and wellness domains (3).

**Domain**		**Definition**
1	Physical	An individual's physical body and may include genetic, environmental, psychophysical, or behavioural components.
2	Mental	Includes cognitive, affective, and behavioural sub-domains.
3	Ethical/spiritual	Ethical awareness, justification of moral beliefs, spirituality (may or may not be aligned with a religious tradition), meaning and purpose.
4	Social/cultural	Larger social circles that individuals participate in such as community (special interest groups, sporting, and cultural groups, etc.)
5	Family	Persons intimately involved in one's life, such as co-habitual partners, dependants, extended family, in-laws, and/or close friends who are perceived as family.
6	Financial	Financial literacy and management ability, and overall net worth.
7	Psychosocial work environment	The design and management of work, and contexts that could impact (positively or negatively) an employee's well-being.
8	Physical work environment	The physical workplace (building, workspace, air quality etc.).
9	Physical home environment	Type and quality of housing one lives in and neighbourhood which impacts safety, access to community services, schools, and important amenities.

While the majority of CMFs manage stressors well, ~10% struggle (4–6) as they navigate military life challenges (e.g., mobility and relocation, risk of injury and illness and absence/separations). Prolonged or cumulative stressors heightens risk of family disruption and poorer health and well-being outcomes (5, 7). These stressors can impact family processes, parent–child attachment and (co)parenting during critical early childhood development (8). SMs/CMFs may benefit from resiliency programs informed by attachment and developmental–ecological frameworks that foster resilience across individual, family, and cultural contexts (8). For example, evidence indicates that resilience can be improved through programs that foster positive attachment and parent-child interactions, provide a safe, structured environment, and shift parenting attitudes and skills (8, 9). Moreover, resilience-training that is strength-based, trauma-informed, and peer-supported, and enables individuals to better navigate and thrive amidst adversity, model resilience, and contribute to building strong and resilient communities, can be powerful.

### Community Resilience

A community's sustained ability to mobilise, respond, and positively adapt to stress (individual, family, community), is affected by its resilience (10, 11). Facilitative environments that increase capacity, provide informal/formal support and resources, and decrease risks, unhealthy communication and trauma can contribute to community resilience (10, 12). According to Patel, core elements of community resilience include local knowledge, community networks and relationships, communication, health, governance/leadership, resources, economic investment, preparedness, and mental outlook (Table 2). Consideration of such elements is important when determining resilience-training programs that might foster community resilience.

**Table 2:** Elements of community resilience as proposed by Patel et al. (12).

**Core element**		**Subthemes**	**Summary description**
A	Local knowledge	- Factual knowledge - Training and education - Collective efficacy and empowerment	Community members must possess an understanding of their potential vulnerabilities and a belief that they are able to overcome hardships.
B	Community networks and relationships	- Connexion - Cohesion	Development of strong links between community members creates a sense of community connectedness and cohesion.
C	Communication	- Effective communication - Risk communication - Crisis communication	Use of common language, meanings and understandings, in addition to the provision of space for individuals to articulate their needs, views, and attitudes allows for open dialogue.
D	Health	- Physical health - Mental health	Understanding and treating physical and mental health needs mitigates additional risks of long-term recoveries after crisis situations.
E	Governance/leadership	- Infrastructure and services - Public involvement and support	Timely and effective responses, along with the involvement of the community for planning responses and recovery increases trust risk and crisis communication.
F	Resources		Appropriate access and allocation of tangible (e.g., food), technical resources (e.g., shelter), and financial and social resources are hypothesised to be connected to community resilience.
G	Economic investment	- Economic programming - Economic development	Proactive investments should ensure interventions are cost-effective and should consider specific needs for a post-disaster community.
H	Preparedness		Individual, family, and government levels must be considered in planning for crisis, prevention, and response and recovery.
I	Mental outlook		Enhancement of mental outlook is conducive toward development of resilient attitudes, feelings, and views in the face of uncertainty, including incorporation of both hope and adaptability.

Within the military, community resilience is built through a “Triad of Responsibility” between the Chain of Command, care provider/team, and SMs/CMFs [(13), p. 11] characterises CAF's approach to health and wellness. Organisationally, CAF deploys tools, programs, and actions to enhance resilience at each stage of a SM's career and provides support for CMFs (1). Program delivery is tasked to service providers (SPs) associated with, for example, CAF Health Promotions [Strengthening the Forces Health Program (14)], military Chaplains (spiritual and familial support for SMs/CMFs), Personnel Support Services (PSP; fitness centres, libraries, camps), and Military Family Resource Centres (MFRCs; social and family services/programs).

A scan, however, of available programs and resources across diverse locations reveals SM/CMF service gaps regarding resilience-building (15).

### CMF Resilience-Building

Within the Canadian military context, potential resilience-training programs for future use with SMs/CMFs would need to be selected and evaluated using criteria such as relevance, efficiency, effectiveness, accessibility, fit, and usability. Other resilience-oriented programs are available in the CAF—most notably the Road to Mental Readiness (R2MR) program. The purpose of the R2MR program is to build awareness of mental illness and operational stress injuries (OSIs) through education, reduce stigma associated with mental illness, and increase understanding and support for these conditions (16). Two complementary made-in-Canada family-focused resilience programs are also being explored. Developed by Kordich-Hall and Pearson (17), Reaching In… Reaching Out (RIRO) is for SPs working with those engaging with parents of young children, and Bounce Back and Thrive! (BBT) is for parents of children 0–8 years of age (18).

RIRO and BBT are standardised, evidenced-informed programs based on the gold standard University of Pennsylvania's Resiliency Program (19). Group psychoeducational peer-supported programs, RIRO and BBT include topics such as fostering calming and focusing, thought-catching and reframing, challenging core beliefs, understanding thinking habits, and generating alternatives (see Table 3 for session topics and objectives of BBT and RIRO). BBT consists of 10, 2-h weekly sessions, while RIRO training is 12-h. RIRO and BBT are trauma-informed, and available in French, English and other languages. The programs have been introduced at one MFRC, utilised with trauma-affected populations, and delivered in person and online. RIRO and BBT have been found to enhance individual and community resilience and foster a culture of resilience (18).

**Table 3:** Reaching In… Reaching Out and Bounce Back and Thrive! Sessions and objectives.

**Reaching In… Reaching Out Section topics**		**Bounce Back and Thrive! Session Topics**	
**Part One** Focus: build foundation of critical resiliency abilities	- Resilience, relaxation, and noticing thoughts - Strengths, resilience, accurate, and flexible thinking - Emotional regulation, self-efficacy, learned control, empathy - Automatic thoughts - Thoughts and feelings - Challenging believes - Generating alternatives - Iceberg beliefs	**Part One** Adult Skills Focus: developing caring relationships and role modelling resilience	- Resilience and family strengths - Role modelling resilience, relaxation and noticing thoughts - Automatic thoughts, reactions and stress - Thoughts and feelings - Thinking habits and managing stressful situations - Healthy and unhealthy beliefs
**Part Two** Focus: application of resiliency skills to children	- Stress and resilience in children - Relaxing, calming, and focusing - Accurate thinking, thinking habits, generating alternatives, and flexible thinking - Developing self-efficacy, positivity, belonging - Thought-feeling connexions, reframing, realistic optimism	**Part Two** Child Application Skills Focus: Facilitating resilience in children	- Empathy - Autonomy, choices, and decision-making - Empowerment - Flexible thinking, hope, and optimism

SPs can receive 40 h of RIRO/BBT trainer training to deliver RIRO and BBT that aims to familiarise them with the material, resources and skills, enhance their understanding of resilience and ability to model resilience, and prepare them to deliver the RIRO and BBT (18).

### Objectives

This study aims to capture the perspectives of SPs post-RIRO/BBT trainer training regarding (1) the impact of the training on their ability to model resilience and deliver RIRO and BBT; and the appropriateness of the RIRO and BBT programs for use with SMs/CMFs and military communities. Specifically, we will determine whether these programs might (2) address service/training gaps regarding resilience-building (3) lend themselves to contextualisation for military populations, and (4) foster a culture of resilience.

## Methods

### Study Design

This study employed a qualitative descriptive research design. Following RIRO/BBT trainer training, audio-recorded focus groups (FGs) were conducted with trainees, the RIRO/BBT Master Trainer and organisational leaders. Ethical approval from a Research Ethics Board and CAF Surgeon General Endorsement were received prior to study initiation.

### Recruitment, Sampling, and Participants

Study participants included SPs (*n* = 20) recruited from a local military base, MFRC, health authority, and University who were invited to participate in RIRO/BBT trainer training due to their roles supporting SMs/CMFs with children 0–8 years of age: MFRCs (*n* = 8; social workers, early child care workers), the Royal Canadian Chaplain Service (RCChS) (*n* = 5; chaplains, mental health chaplains), CAF transition unit (*n* = 1, SM), a family (*n* = 1; parent), the local health authority (*n* = 1; social worker), university-based researchers/clinician-scientists (*n* = 4; nurse, occupational therapist, psychologist, social worker). Organisational leaders from the MFRC (*n* = 2) and RCChS (*n* = 1), and the RIRO/BBT Master Trainer (*n* = 1), also participated in FGs.

### Intervention

RIRO/BBT trainer training was delivered in-person at the local MFRC for 5 days (September 9–13, 2019) by a RIRO/BBT Master Trainer to 20 trainees. RIRO-specific training occurred on Days 1–3 and BBT training on Days 4–5.

### Data Collection

Data were collected during six FGs following RIRO/BBT trainer training using semi-structured interview questions. FG questions were based on study objectives and purposely broad and iterative so as to facilitate discussion (20). Questions included, “What was your experience of the program? What aspects of the program were the most/least helpful/impactful/effective? How might the program be improved/contextualised for CMFs?” FGs were facilitated by three research team members with 10 years' experience conducting FGs, while a fourth member recorded field notes. The facilitators actively engaged participants and encouraged sharing of opinions and experiences, while the observer monitored engagement, participation, and verbal and non-verbal cues (21, 22). Qualitative analysis occurred during and following the FGs through reflection and discussion (21, 22).

A FG with trainees and the Master Trainer was held at the end of training Day 5 (FG1; *n* = 21), followed in the 2 weeks thereafter with MFRC and RCChS leadership (FG2; *n* = 3), and a CMF member, transition unit SM, and research team members (FG3; *n* = 5). Organisation-specific FGs were conducted with trainees later in September and October 2019 (FG4 and FG5; *n* = 20) and in November 2020 (FG6; *n* = 3). In instances where participants were unable to attend due to operational commitments, participants provided feedback by email. The FGs were conducted in a private setting at the MFRC, lasted ~2 h, and were digitally recorded and transcribed verbatim by a transcriptionist. As participant identifiers were intentionally removed during the transcription process, quotes are identified by FG rather than participant.

### Data Analysis

The FG data were analysed using thematic analysis as described by Braun and Clarke (23). Braun and Clarke (23) argued that thematic analysis provides a flexible method for identifying, analysing, and reporting themes in rich detail. To ensure the validity, reliability, and conformability of the analysis, the data were thematically-analysed (inductively and deductively) by four research team members. This also ensured inter-rater reliability and bracketing of researcher bias (24). An audit trail was used to review and examine decisions and maintain credibility and rigour (25).

Initial coding was completed independently and by hand by two team members who had not conducted the FGs. Initial inductive open codes were then combined into preliminary themes focused on similarities and differences within and between FGs. Preliminary themes then underwent a secondary round of collective analysis by two other team members focused on deductive coding informed by study objectives, resilience domains, core community resilience elements (12) and the aforementioned evaluation criteria. Regular meetings enabled discussion, code verification, resolution of discrepancies and determination of final themes and supporting quotes.

## Results

Thematic analysis revealed several salient themes related to participants' experiences: (1) RIRO/BBT trainer training enhanced SPs' abilities to model resilience and deliver RIRO and BBT programs. RIRO and BBT programs (2) are useful for families and communities, (3) could assist in developing resilient environments, and (4) could be adapted for use with military populations. Themes support several of the total health and wellness domains and core elements of community resilience, with the potential for enhancement of local knowledge, community networks and relationships, communication, preparedness, and mental outlook being evident in participant feedback. Further discussion of themes follows, in addition to a table of prominent themes, subthemes, and supportive quotes, resilience domain and core elements (Table 4).

**Table 4:** Themes, subthemes, and supportive quotes and resilience domains/core elements.

**Theme**	**Subtheme**	**Supportive quote**	**Resilience domains (1–9) and core elements (A–I)**
Service provider capacity-building	Enhances role modelling	“*[B]ecause we are such a small community, if we can be role modelling it more often they will see the results of it.” (FG4)*	2, 3, 4, 5, 7 A, B, C, I
	Offers useful, simple, applicable and understandable tools	“*I found with this it was greatly expanded on the levels and the amount of skills [provided in other training] and I think that is good.” (FG5)“[R]esiliency is such a catchword (*…*) So what does that mean? You know you can explain it but in practical terms this program gives you practical applications of what that looks like.” (FG4)*	2, 4, 5, 7 A, B, C, H, I
	Increases personal and professional awareness of thoughts and behaviours	“*At least in my units, our units, we are dealing with a younger population in general. The body of folks are privates or corporals or master corporals and those tend to be between the ages of 17–25 so they are first time parents, or… first time out the door with deployments so any kind of CBT skill sets that can be modeled and or explained and done is really helpful; not only personally, but in their professionalism as well, because they encounter a lot of stressful situations and then fall into the same patterns of thought.” (FG5)*	2, 4, 5, 6 A, B, C
	Offers a mechanism for SP capacity-building	“*I think it would be fantastic if we could have more individuals [trained]. So if we had had somebody from PSP who's been able to do the training for people who are coming to do camps (*…*) [I]t might be worthwhile to have some additional people within the community who are able to provide the facilitator role or be a facilitator.” (FG6)*	4, 5, 7 A, B, C
Community and family application	Provides helpful skills and strategies	“*As a parent of [toddlers] (*…*) I found the breathing and counting exercises helpful to help my son self-regulate. (*…*) When my son is having a temper tantrum he is trying to tell me something. That one insight has changed how I respond to him.” (P1)*	2 A, C
	Supports family building	“*[I] think it is huge for the spouses, and their resilience with just the high tempo of our soldiers right now. Because a happy home does mean a happy soldier. (*…*) We could just introduce it there… I think you get the most bang for your buck. I think things will really permeate into our soldiers lives as well.” (FG5)*	2, 5 A, B, I
	Enhances understanding of behaviours	“*I think it would be wonderful to be part of that pre-deployment window and also the post deployment window (*…*) when they come back they are super excited that the partner is back but that is when the rub starts to happen, when they are not meshing like they did necessarily before they went away so to learn and to re-emphasise some of that skill set when the members come back from deployment I think would be very useful.” (FG5)*	2, 4, 5, 6 B, C
Resilient environments	Supports community resilience-building	“*[W]hen we talk of deployments they are gone then they are back. They are constantly leaving and reintegrating. And their family dynamics*… *they are not consistent. They are always in flux. People are constantly adjusting so I think keeping that and adding that to this would fit be perfect, because it is all about keeping resilient.” (FG4)*	2, 4, 5, 7 A, B, C, H, I
	Cultivates a common language	“*I think that somewhat of a common language (*…*) that helps too. Like a lot of the kids don't have a lot of consistency so then at least having consistency at their school or the daycare they go to and then this program that their Mom knows a couple of things.” (FG4)* “*[T]hat's the whole idea of RIRO—a community approach to resilience so that everybody is using similar language; everybody has a similar understanding of the development and strengthening of resilience” (FG6)*	2, 4, 5 A, B, C
	Benefits the community	“*[T]he base is its own community (*…*) We run a daycare and out of school care. (…) We have a school here. So a lot of people are interacting with kids on our base. (*…*) There's a lot of day homes (*…*) [T]hey (*…*), our community and our kids would benefit from the facilitation of [RIRO and BBT].” (FG6)*	4, 5, 7 B, C
Usability	Is culturally adaptable/sensitive	“*Maybe just bringing in some of the common themes of military life like deployment is a big one, like those challenges even to bring in example*… *so it is not the program you are changing but just the way that people understand it. (*…*) [F]ocus on what constant disruption [and isolation] looks like.” (FG4)*	4, 5, 7 A, B, C, H
	Lends itself to flexible program delivery	“*I think a more intensive experience a whole day or whatever with two Saturdays (*…*) you are going to have more buy in just it will work better than 10 weeks” (FG5)*	5 A, B
	Enriches community outreach	“*[Reaching out to] some of the places where there are more concentrated numbers. training some of the daycares there (*…*) [W]e cannot necessarily train every single [local] agency, but maybe the ones we know there are more concentrated [Military] kids. We had some schools asking for services, so they might be teachers we want to train so they develop the skills.” (FG4)*	2, 4, 5, 7 A, B, H, I

### Theme I: Service Provider Capacity-Building

#### Resilience Role-Modelling

Participants noted that resilience programs fostering adult modelling of resilience skills is currently lacking across bases and MFRCs. RIRO/BBT trainer training inspired them to more intentionally model resilience in their homes and workplaces: “*[B]ecause we are such a small community, if we can be role modelling it more often they will see the results of it.”* (FG4)

#### Resilience Tools

Participants found that the training offered relevant, useful, simple, applicable and understandable tools across resilience domains (i.e., physical, mental, ethical/spiritual, social/cultural, familial, financial) and the psychosocial work environment that, “*everybody could use.”* (FG5) The training expanded their knowledge and offered ways to alter tools, including using “*[An aide memoire] like a cheat sheet or a quick refresher that you carry with you (*…*) the first page could be the 3Rs [or another learned tool] (*…*) After a while it becomes muscle memory and you don't need it.”* (FG1) “*[A]ctive breathing (*…*) is not necessarily a skill that everyone wants or practises (*…*) Fair enough. Here is another thing you can try.”* (FG5) They also identified times, such as pre- and post-deployment, during which increased resilience skills may support CMFs, younger CAF members, and SMs across all ranks.

#### Personal Awareness

Participants indicated that the training enhanced their self-awareness, “*Personal awareness… to understand my strengths and weaknesses. In terms of understanding different styles of thinking,”* (FG5) and their own parenting: “*[I]t was helpful to (*…*) pause and spend time thinking about your reaction as a parent and thinking about the children's reactions, and how that maybe we are not managing them in the best way.”* (FG5)

#### Professional Awareness

The training offered participants an opportunity to gain insight into areas that they and others may need to develop: “*I am trying to understand what might be struggles for people, (*…*) [and how and] when (*…*) to help (*…*) people improve their resiliency and (*…*) see that in-depth journey.”* (FG5) It also equipped them with new strategies to “*approach some topics… to help families and the members, (*…*) understand thought traps [thinking habits].”* (FG5)

#### Mechanism for SP Capacity Building

Participants saw benefit to involving SPs from various community partners to ensure widespread use of skills: “*[I]t would be fantastic if we could have (*…*) additional people within the community who are able to (*…*) be a facilitator.”* (FG6)

### Theme II: Community and Family Application

#### Skills and Strategies

Participants believed that the training could “*[H]elp [SMs] to recognise their emotions, how they think and then give them the necessary tools to correct and adjust their behaviour and thought processes.”* (P1) They recommended that the tools be taught in basic training as it would, “*[S]et a healthy thought pattern and provide each member with coping skills.”* (P1) Another participant noted, “*Chapters 1–3 of RIRO should be mandatory for all ranks. The information in it is probably some of the best building blocks I've seen.”* (FG3) In addition to providing regulation tools, they recognised the impact of equipping adults with resilience skills that can be used across all domains with their children and across the lifespan.

#### Family-Building

Strengthening CMFs is important to developing community resilience. Participants related that BBT supports family-building as it is, “*[V]ery family-oriented (*…*) and is presented in a language that speaks well to that demographic.”* (FG6) It could be useful in “*[T]eaching parents to know themselves and self-regulate. And then in turn help their kids to self-regulate and express when they are having difficulty.”* (FG5)

#### Role Modelling

Participants noted improvements in their own families as a result of role modelling, “*[A]s my wife would say, I often would (*…*) dad-splain (*…*) to help [my children] through whatever they are feeling. (*…*) [RIRO] provided me with a language and the avenue for engagement with my children that I was lacking (*…*) and it has made a very positive impact on my relationship with my kids.”* (FG5)

#### Behaviours

Participants identified specific periods of time, such as pre- and post-deployment windows, where increased resilience skills may be beneficial in supporting families to understand patterns of behaviour: “*[I]t would be wonderful (*…*) pre-deployment (*…*) and (*…*) post deployment (*…*) when they are not meshing like they did necessarily before they went away.”* (FG5)

### Theme III: Resilient Environments

#### Community Resilience-Building

“*[The CMF community] is the environment that the kids are in and the people who are with them all the time.”* (FG6) As community members often support each other amidst frequent change, uncertainty, relocation, deployments, extended absences of a parent/caregiver, and lack of SP and program continuity from one location to the next, equipping CMF communities with resilience tools is essential. Participants noted that families have been struggling to cope with the increased tempo of operations, indicating that, “*[W]e are to support our families to figure out tools to help them to survive the military profession or at least until the tempo comes down and they are not so busy.”* (FG5) Participants noted that access to resilience programs addressing adult modelling of resilience skills to children and child resilience skill-building is currently needed across bases and MFRCs: “*[C]hildren are constantly adjusting. (*…) *So building up resiliency skills for children is a good thing.”* (FG4)

#### Common Language

The benefits that standard evidence-based resilience programs offer regarding use of a common language were also noted: “*It would be good to have all the staff trained in it so we have the same style or the same verbiage with children that come into our care. Or even families that we're trying to help them build their resiliency skills.”* (FG4)

#### Community Benefits

Participants recognised the benefits that RIRO/BBT trainer training might have for other SPs, “*[PSP] staff at the library, (*…*) they see enough [children/CMFs] that it would be great to have some of those people [trained]”* (FG6), and the ways in which RIRO and BBT might complement other programs: “*We have health promotions here. They run a lot of programs. [T]here's bits and pieces (*…*) but not a complete program like [RIRO/BBT].”* (FG6) RIRO and BBT appear to align with existing CAF and MRFC program offerings and would address a service gap.

### Theme IV: Usability

#### Cultural Adaptability

Participants noted that RIRO and BBT content would need to be contextualised for the military populations: “*[T]he language, (*…*), some of the examples, need to change for it to communicate better to our soldiers.”* (FG5) “*Examples I think would be worthwhile because it may be easier for someone to pick it up, but outside of that they've tested this all across trauma, significant trauma communities. Why would we be any different? (*…*) It's the nominal changes to update the program vs. militarise it.”* (FG2). Additionally, participants noted that, “*[A]ctivities could be altered so they are a little bit more (*…*) military-friendly.”* (FG4) Changes training content and material to neutralise gender could also be helpful: “*If men are in the group, some male perspectives, changing the colour scheme might be helpful, because for me, it's feminine to a core (*…*) some guys might be turned off by that.”* (FG1) It was further noted that, “*[I]f [RIRO] doesn't emphasise separations, deployments*… *it will just be seen as another [civilian] course that is trying to make its way into a military context.”* (FG5).

Some participants cautioned against too much militarization during contextualisation as, “*People are people. When you grieve because your dad died, it doesn't matter that I wear a green uniform (*…*) I grieve the same way whether I wear a green uniform or I don't, but ‘we have to put it in the context because we’re separate across the country' but a lot of people are separate across the country.”* (FG2) Additionally, “*Some spouses want nothing to do with the military, so if you give them too many military examples they won't get it. So it's making sure there's enough of both.”* (FG2)

#### Program Delivery

Participants noted a need for reduced time commitment, “*We have to summarise it to make it in a military context. It might be hard to get the people 1 week off from what they do.”* (FG5) Participants recommended providing condensed or intensive versions of RIRO and BBT. “*My concern is the longevity. Ten weeks is a long time. I kind of wish it were 6 or 8.”* (FG1) Despite the length, participants indicated, “*From an educator perspective, the lesson plans are very clear, could be delivered in a simplistic manner. (…) [Y]ou could open up the book and know what you needed to teach.”* (FG3)

#### Community Collaboration and Outreach

Participants suggested several potential collaboration options: “*Some units have a family cell so finding out who the member is that runs that cell within a unit is a good connexion.”* (FG4) “*Sentinels (peer supports embedded) in the various units. RIRO offers useful tools to help them help others,”* (FG5) and, “*We do have a school liaison coordinator who (*…*) may be a good person to get that out to the school newsletters.”* (FG4) The RIRO and BBT programs could be expanded to other SPs throughout the CAF and MFRCs.

## Discussion

The development of resilient SMs/CMFs is essential to fulfiling the vision and priorities of CAF and VAC. Offering resilience training to SPs and SMs/CMFs within the CAF community and through MFRCs can foster resilient behaviours, attitudes, and mindsets, enhance individual, family and community resilience and have intergenerational impacts. Currently, evidence-informed resilience-building programs that foster resilience within the CAF, VAC, and MFRC communities are lacking.

Trainees related that the RIRO and BBT resilience training programs, with their holistic, integrated, experiential learning and community approach, were likely to facilitate resilience-building in military and CMF communities. Skills and tools crossed resilience domains (i.e., physical, mental, ethical/spiritual, social/cultural, familial) and the psychosocial work environment. The programs also addressed many core elements of community resilience (i.e., local knowledge, community networks and relationships, communication, preparedness, and mental outlook). Participants perceived program content to be relevant, effective, useful, and accessible. Alignment and fit with CAF and VAC initiatives was also noted, although contextualisation and inclusion of military-specific scenarios would greatly enhance usability and reflect the lived experiences of SMs/CMFs. Trainees indicated that the programs would address a service gap and be complementary to current CAF and MFRC programs.

### Capacity-Building

Intentional-capacity building across SPs, SMs/CMFs, MFRCs, and the CAF, together with clear and supported implementation efforts, would be required to integrate resilience-building programs such as RIRO and BBT in military, Veteran and CMF populations. Selection and contextualisation of a particular program(s), facilitator training, and embedding of training into existing program offerings and routine training cycles would also be needed. Master trainers, authorized to train others in the delivery of RIRO and BBT, would need to be credentialed to support widespread program implementation. It would also be of benefit for sufficient numbers of SPs and SMs/CMFs (25%) to receive RIRO and BBT resilience training so as to enhance the likelihood of effecting culture change at the individual, family and organisational levels (26). Making the program available in differing formats (in-person, online), length, and language specific to target audiences would also enhance uptake. This would be especially helpful for reaching SMs and CMFs who reside off base [currently 85% of CMFs (27)], or are geographically dispersed, living abroad, or separated due to duty-related activities. Online delivery would make it possible for simultaneous program participation by co-parents. Virtual RIRO/BBT peer support groups would also have the benefit of facilitating community connexion and resilience. Overarching resilience networks and hubs could create a greater sense of community and increase capacity and support, allowing SMs, Veterans, CMFs and communities to flourish during and following life in military service.

### Future Research

This study lays a foundation for future research regarding resilience-training programs and capacity-building within a Canadian military context. It also contributes to the much-needed evidence-base regarding culturally-specific and sensitive resilience-building programs. Further research is yet needed to assess the impact of RIRO and BBT training on SPs, SMs and Veterans, CMFs, and the CAF at large, and the effectiveness of RIRO and BBT. Consideration of programs for CMFs with older children and family members is also needed. Additionally, the implementation, spread and sustainability of RIRO and BBT programs in the CAF and VAC communities, and establishment of community resilience networks and hubs, has not been explored. Various training and program delivery formats (online and in-person formats in various languages) is also warranted.

### Strengths and Limitations

This study had several strengths and limitations. Participation of multidisciplinary SPs and leaders who support SMs/CMFs and are familiar with the military culture, as well as SMs themselves, was a strength, as was capturing trainee perspectives immediately upon completion of RIRO/BBT trainer training. Limitations include the small sample size and lack of VAC representation, although SPs working specifically with Veterans were included. Findings were also based on discussions with the trainees prior to them delivering RIRO and BBT programs, with the exception of FG6 participants who delivered BBT training to a sample of CMFs. Additionally, not all participants who attended the RIRO and BBT training were able to join the FGs, and instead sent email responses to the questions; these responses may have been less detailed and rich. Further, due to the unforeseen circumstances of a global pandemic (COVID-19), exploration of opportunities and plans for RIRO/BBT trainees to offer either RIRO or BBT programs was not possible.

## Conclusion

The resilience and operational readiness of SMs/CMFs is essential to CAF's ability to fulfil its missions. Resilience-training can enhance the resilience of SPs and SMs/CMFs and military communities. Preparing SPs to model resilience and deliver evidenced-based resilience-training programs such as RIRO and BBT has the potential to build capacity at the individual, family, community, SP and organisational levels, and support a resilient and ready defense team. Widespread use of standardised resilience programs could enable use of a common language and skill set in the military community and growth of a culture of resilience. This study contributes to research regarding evidence-based culturally-specific and -sensitive resilience-training programs and capacity-building within the Canadian military context.

## Data Availability Statement

The raw data supporting the conclusions of this article will be made available by the authors, without undue reservation.

## Ethics Statement

The studies involving human participants were reviewed and approved by University of Alberta Research Ethics Board. The patients/participants provided their written informed consent to participate in this study.

## Author Contributions

SB-P, AP, AH, JO, PS, and CM conceived, designed, and conducted the study. All authors drafted, revised, and approved the final manuscript submitted for publication.

## Conflict of Interest

CM was employed by the MFRC. The remaining authors declare that the research was conducted in the absence of any commercial or financial relationships that could be construed as a potential conflict of interest.
